# Phase 3 Study of Talazoparib Plus Enzalutamide Versus Placebo Plus Enzalutamide as First‐Line Treatment in Patients With Metastatic Castration‐Resistant Prostate Cancer: TALAPRO‐2 Japanese Subgroup Analysis

**DOI:** 10.1002/cam4.70333

**Published:** 2024-12-31

**Authors:** Nobuaki Matsubara, Hideaki Miyake, Hiroji Uemura, Atsushi Mizokami, Hiroaki Kikukawa, Takeo Kosaka, Kazuo Nishimura, Motonobu Nakamura, Kazuki Kobayashi, Atsushi Komaru, Yuko Mori, Shigeyuki Toyoizumi, Natsuki Hori, Yoshiko Umeyama, Hirotsugu Uemura

**Affiliations:** ^1^ National Cancer Center Hospital East Kashiwa Japan; ^2^ Hamamatsu University School of Medicine Hamamatsu Japan; ^3^ Kobe University Graduate School of Medicine Kobe Japan; ^4^ Yokohama City University Medical Center Yokohama Japan; ^5^ Kanazawa University Hospital Kanazawa Japan; ^6^ Kumamoto Medical Center Kumamoto Japan; ^7^ Keio University Hospital Tokyo Japan; ^8^ Osaka International Cancer Institute Osaka Japan; ^9^ Kyushu Cancer Center Fukuoka Japan; ^10^ Yokosuka Kyosai Hospital Yokosuka Japan; ^11^ Chiba Cancer Center Chiba Japan; ^12^ Pfizer R&D Japan Tokyo Japan; ^13^ Kindai University Hospital Osaka Japan

**Keywords:** enzalutamide, Japanese, mCRPC, PARP inhibitor, TALAPRO‐2, talazoparib

## Abstract

**Background:**

In TALAPRO‐2, the poly(ADP‐ribose) polymerase inhibitor talazoparib plus the androgen receptor–signaling inhibitor enzalutamide improved radiographic progression‐free survival (rPFS) versus placebo plus enzalutamide (hazard ratio [HR] = 0.63; 95% CI, 0.51–0.78) in molecularly unselected patients with metastatic castration‐resistant prostate cancer (mCRPC). We report an exploratory analysis of efficacy, safety, and pharmacokinetics in Japanese patients enrolled in the TALAPRO‐2 study.

**Methods:**

The ongoing, multinational, randomized, double‐blind, phase 3 TALAPRO‐2 study enrolled patients with mCRPC receiving ongoing androgen deprivation therapy. Patients were prospectively assessed for homologous recombination repair (HRR) gene alterations and randomized 1:1 to receive talazoparib or placebo plus enzalutamide once daily. The primary endpoint was rPFS by blinded independent central review (BICR). Secondary endpoints included overall survival, objective response, safety, and pharmacokinetics.

**Results:**

For the 116 Japanese all‐comers patients enrolled in TALAPRO‐2, the HR for rPFS was 0.89 (95% CI, 0.45–1.75) for the talazoparib versus placebo arm; among those with HRR‐deficient disease, the HR was 0.58 (95% CI, 0.16–2.20). Among patients with *BRCA1/2* gene alterations in the HRR‐deficient population (*n* = 10), the HR for rPFS was < 0.01 (95% CI, < 0.01–not reached) for the talazoparib versus placebo arm. In the all‐comers population, the objective response rate by BICR was 55% (all complete responses) in the talazoparib arm versus 36% in the placebo arm. The safety profile of talazoparib plus enzalutamide was similar between Japanese patients and the overall all‐comers population; no new safety signals were identified. Anemia was the most common grade 3/4 treatment‐emergent adverse event (55%) and cause of talazoparib discontinuation (12%). Talazoparib *C*_trough_ was comparable across Japanese, Asian, and non‐Asian subgroups.

**Conclusions:**

In this exploratory analysis, efficacy outcomes with talazoparib plus enzalutamide in Japanese patients in TALAPRO‐2 were consistent with those in the overall all‐comers population. The safety profile and pharmacokinetics of the combination were similar between Japanese patients and the overall all‐comers population.

**Trial Registration:** ClinicalTrials.gov Identifier: NCT03395197

## 1 Introduction

Despite recent advances in the treatment of metastatic castration‐resistant prostate cancer (mCRPC), it remains incurable, necessitating new therapies [1, 2]. Many preclinical and clinical studies suggest that combining an androgen receptor–signaling inhibitor (ARSi) with a poly(ADP‐ribose) polymerase (PARP) inhibitor (PARPi) may extend the efficacy of PARPi to prostate tumors both with and without homologous recombination repair (HRR) gene alterations [3, 4, 5, 6]. Therefore, coinhibition of both the androgen receptor and PARP may result in enhanced benefit in tumors regardless of HRR gene alteration status [4].

TALAPRO‐2 (NCT03395197) is an ongoing phase 3 study evaluating talazoparib, a potent PARPi, plus enzalutamide, an ARSi, as first‐line treatment in patients with mCRPC [6, 7]. Results from TALAPRO‐2 showed a significant improvement in the primary endpoint of radiographic progression‐free survival (rPFS) by blinded independent central review (BICR) and a favorable trend toward improved overall survival in patients who received talazoparib plus enzalutamide versus placebo plus enzalutamide in both a molecularly unselected population (all‐comers) and an HRR‐deficient population [6, 8]. Anemia was the most common grade 3/4 treatment‐emergent adverse event (TEAE) in both the all‐comers and the HRR‐deficient populations [6, 8]. Efficacy and safety results from TALAPRO‐2 supported the recent approval of talazoparib plus enzalutamide by the United States Food & Drug Administration for treatment of mCRPC with HRR gene alterations, and by the Japanese Pharmaceuticals and Medical Devices Agency for treatment of mCRPC with *BRCA1/2* gene alterations and distant metastases [9, 10].

In East Asia, diagnoses of prostate cancer have increased in recent years [11]. In 2022, prostate cancer was the most commonly diagnosed cancer in Japanese men with 104,318 cases reported; this accounted for 18% of all cancer diagnoses and was the sixth most common cause of death due to cancer (6% of all cancer deaths) [12]. The efficacy and safety of therapeutic agents can vary between Asian and Western populations possibly due to environmental and genetic differences [13, 14], and thus provide a rationale for analyzing outcomes in Japanese patients who participated in the TALAPRO‐2 study to investigate whether they are similar to the overall population. Based on two separate phase 1 trials of talazoparib monotherapy, the recommended dose (1 mg once daily [QD]) was the same for both Japanese and non‐Japanese patients [15, 16]. However, the initial nonrandomized open‐label run‐in of TALAPRO‐2 demonstrated similar talazoparib exposure levels when 0.5 mg talazoparib was administered in combination with enzalutamide compared with 1 mg talazoparib given as monotherapy [6]. Therefore, patients in TALAPRO‐2 received a starting dose of 0.5 mg talazoparib (0.35 mg QD for moderate renal impairment) plus 160 mg enzalutamide QD [6, 8]. Here, we report the efficacy, safety, and pharmacokinetics of talazoparib plus enzalutamide versus placebo plus enzalutamide in Japanese patients enrolled in TALAPRO‐2, and compare outcomes with the overall population [6, 8].

## 2 Patients and Methods

### 2.1 Study Design and Participants

The TALAPRO‐2 study design and details of patient enrollment have been previously described [6, 8, 17]. TALAPRO‐2 is an ongoing, multinational, randomized, double‐blind study that enrolled patients aged ≥ 18 years (≥ 20 years in Japan) with asymptomatic or mildly symptomatic mCRPC. Eligible patients were receiving ongoing androgen deprivation therapy, had an Eastern Cooperative Oncology Group performance status (ECOG PS) of 0 or 1, had progressive disease at study entry, had adequate bone marrow function (e.g., hemoglobin ≥ 9 g/dL), and were naïve to life‐prolonging systemic therapy for CRPC. Previous abiraterone, orteronel, or docetaxel in the castration‐sensitive setting were allowed. Patients were randomized 1:1 to receive either talazoparib plus enzalutamide QD (talazoparib arm), or placebo plus enzalutamide QD (placebo arm) (Figure S1). Randomization was stratified by prior second‐generation androgen receptor pathway inhibitor (abiraterone or orteronel) or docetaxel in the castration‐sensitive setting (yes vs. no). In the all‐comers population only, randomization was stratified by HRR gene alteration status (deficient vs. nondeficient or unknown).

Prior to randomization, patients consented to provide solid tumor tissue and/or blood‐based samples that were prospectively assessed using FoundationOne®CDx and/or FoundationOne®Liquid CDx (Foundation Medicine, Cambridge, MA, USA) for the following HRR gene alterations: *BRCA1*, *BRCA2*, *PALB2*, *ATM*, *ATR*, *CHEK2*, *FANCA*, *RAD51C*, *NBN*, *MLH1*, *MRE11A*, and *CDK12*. Patients were initially enrolled into an all‐comers cohort unselected for HRR gene alterations (*N* = 805; *n* = 169 patients had HRR gene alterations). The 169 patients with HRR gene alterations were combined with an additional subsequently enrolled group of 230 patients with HRR gene alterations to form the combined HRR‐deficient population (*N* = 399; Figure S1).

### 2.2 Outcomes

Primary and secondary endpoints for TALAPRO‐2 have been previously reported [6, 8, 17]. The primary endpoint in TALAPRO‐2 was rPFS by BICR per Response Evaluation Criteria in Solid Tumors (version 1.1; soft tissue disease) and Prostate Cancer Clinical Trials Working Group 3 (bone disease). Secondary endpoints included overall survival (OS), investigator‐assessed rPFS, confirmed objective response rate (ORR), time to prostate‐specific antigen (PSA) progression, time to initiation of cytotoxic chemotherapy, time to disease progression or death on the first subsequent antineoplastic therapy for prostate cancer by investigator assessment (PFS2), safety (graded by National Cancer Institute Common Terminology Criteria for Adverse Events [18] version 4.03), and pharmacokinetics (not yet reported). Exploratory analyses for the Japanese subgroup included rPFS (by BICR and investigator), OS, ORR, time to PSA progression, time to initiation of cytotoxic chemotherapy, PFS2, safety, and pharmacokinetics.

### 2.3 Statistical Analysis

Full details of the statistical analysis for TALAPRO‐2 have been described elsewhere [6, 8, 17]. Briefly, time‐to‐event endpoints were compared between treatment arms using a stratified log‐rank test unless otherwise stated. HRs and associated 95% confidence intervals (CIs) were estimated by Cox proportional hazards model. Median time‐to‐event endpoints were estimated by the Kaplan–Meier method, and 95% CIs were based on the Brookmeyer–Crowley method. SAS version 9.4 statistical software was used for all analyses. All reported *p* values are two sided. The analysis of the Japanese subgroup did not have formal statistical power because of its post hoc, exploratory nature.

### 2.4 Role of the Funding Source

The sponsor (Pfizer) was involved in the trial design (together with the academic steering committee), data analysis, and data interpretation, and funded medical writing support. Astellas Pharma, Inc. provided enzalutamide. All authors, including those employed by the sponsor, contributed to data interpretation as well as development, writing, and approval of the manuscript.

## 3 Results

### 3.1 Demographic Characteristics of Patients

A total of 131 Japanese patients were enrolled in TALAPRO‐2; in the overall all‐comers population, 116 (14%) of 805 patients were Japanese (Figure S1). Among patients randomized to talazoparib plus enzalutamide or placebo plus enzalutamide, 60 (15%) of 402 and 56 (14%) of 403 were Japanese, respectively. Baseline characteristics of the Japanese subgroup and the overall all‐comers population were well‐balanced between the treatment arms (Table 1). While median age was similar between the Japanese subgroup and the overall all‐comers population, Japanese patients had a lower median weight and median PSA; a higher proportion of Japanese patients had an ECOG PS of 0, and a lower proportion of Japanese patients had received prior docetaxel (Table 1). In the Japanese subgroup, 24 (21%) of 116 patients were HRR‐deficient (169/805 [21%] in the overall all‐comers population). The rates of alterations in specific HRR genes were comparable between the Japanese subgroup and the overall all‐comers population and consistent with prior reports [19, 20, 21] (Table 1).

**TABLE 1:** Baseline demographics and disease characteristics (all‐comers intent‐to‐treat population).

	Japanese subgroup		Overall all‐comers population	
	Talazoparib + enzalutamide (*n* = 60)	Placebo + enzalutamide (*n* = 56)	Talazoparib + enzalutamide (*N* = 402)	Placebo + enzalutamide (*N* = 403)
**Age, median (range), years**	72 (44–87)	73 (50–85)	71 (41–90)	71 (36–91)
**Weight, median (range), kg**	67 (54–102)	68 (50–98)	79 (45–169)	81 (48–178)
**PSA, median (range), ng/mL**	7.9 (0.3–460.0)	8.2 (0.6–268.0)	18.2 (0.1–2796.0)	16.2 (0.1–2285.1)
**Gleason score,** ***n*** **(%)** ^a^				
< 8	10 (17)	4 (7)	117 (29)	113 (28)
≥ 8	50 (83)	52 (93)	281 (70)	283 (70)
**Disease site,** ***n*** **(%)**				
Bone (including with soft tissue component)	52 (87)	50 (89)	349 (87)	342 (85)
Lymph node	17 (28)	20 (36)	147 (37)	167 (41)
Visceral (lung)	11 (18)	6 (11)	45 (11)	61 (15)
Visceral (liver)	2 (3)	1 (2)	12 (3)	16 (4)
Other soft tissue	2 (3)	0	37 (9)	33 (8)
**ECOG PS,** ***n*** **(%)**				
0	55 (92)	53 (95)	259 (64)	271 (67)
1	5 (8)	3 (5)	143 (36)	132 (33)
**Prior abiraterone** ^b^ **or docetaxel,** ***n*** **(%)** ^**c**^	8 (13)	4 (7)	109 (27)	110 (27)
Abiraterone^d^	3 (5)	4 (7)	21 (5)	25 (6)
Docetaxel^d^	5 (8)	1 (2)	86 (21)	93 (23)
**T** **issue source for prospective HRR gene alteration testing,** ***n*** **(%)**				
Tumor tissue	47 (78)	45 (80)	345 (86)	345 (86)
Tumor tissue and blood (circulating tumor DNA)	13 (22)	11 (20)	57 (14)	58 (14)
**HRR gene alteration status by randomization stratification,** ***n*** **(%)**				
Deficient	13 (22)	11 (20)	85 (21)	84 (21)
Nondeficient or unknown	47 (78)	45 (80)	317 (79)	319 (79)
**HRR gene alteration status by prospective tumor tissue and blood testing,** ***n*** **(%)**				
Deficient	13 (22)	11 (20)	85 (21)	82 (20)
Nondeficient	25 (42)	35 (62)	207 (51)	219 (54)
Unknown	22 (37)	10 (18)	110 (27)	102 (25)
**Number of patients with HRR gene alterations, *n* (%)**				
One or more alterations in the corresponding gene	13 (22)	11 (20)	85 (21)	82 (20)
*ATM*	5 (8)	2 (4)	23 (6)	14 (3)
*BRCA1*	0	0	5 (1)	4 (1)
*BRCA2*	3 (5)	4 (7)	23 (6)	28 (7)
*CDK12*	4 (7)	5 (9)	23 (6)	29 (7)
*CHEK2*	1 (2)	0	6 (1)	5 (1)
Other^e^	2 (3)	1 (2)	14 (3)	13 (3)

Abbreviations: ECOG PS, European Cooperative Oncology Group performance status; HRR, homologous recombination repair; PSA, prostate‐specific antigen.

^a^ Not reported for the remaining patients.

^b^ Overall population: two patients in each treatment arm received prior orteronel.

^c^ Number of patients by interactive web response system.

^d^ Number of patients obtained from the clinical database.

^e^ Japanese subgroup includes *MLH1*, *MRE11A*. Overall population includes *ATR*, *FANCA*, *MLH1*, *MRE11A*, *NBN*, *PALB2*, *RAD51C*.

In the HRR‐deficient population, 39 (10%) of 399 patients were Japanese, with 22 (11%) of 200 in the talazoparib arm and 17 (9%) of 199 in the placebo arm (Figure S1). Of these Japanese patients, 4 (18%) of 22 in the talazoparib arm and 6 (35%) of 17 in the placebo arm had *BRCA1/2* gene alterations. In the overall HRR‐deficient population, 71 (36%) of 200 patients and 84 (42%) of 199 patients in the talazoparib and the placebo arms had *BRCA1/2* gene alterations, respectively.

### 3.2 Efficacy

**FIGURE 1:**
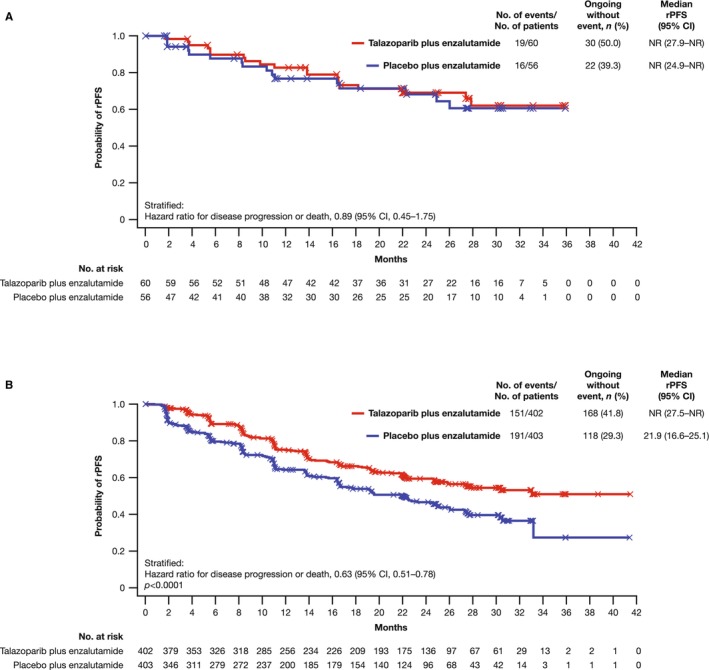
rPFS by BICR: (A) Japanese subgroup; (B) overall population (all‐comers intent‐to‐treat population). BICR, blinded independent central review; CI, confidence interval; rPFS, radiographic progression‐free survival. To maintain the overall type I error at or below one‐sided 0.025, alpha for rPFS by BICR was split equally between the all‐comers and the HRR‐deficient populations (one‐sided alpha of 0.0125 for each). Panel B is reprinted from *The Lancet*, Volume 402, Agarwal et al., Talazoparib plus enzalutamide in men with first‐line metastatic castration‐resistant prostate cancer (TALAPRO‐2): A randomized, placebo‐controlled, Phase 3 trial, pages 291–303, 2023, with permission from Elsevier.

**FIGURE 2:**
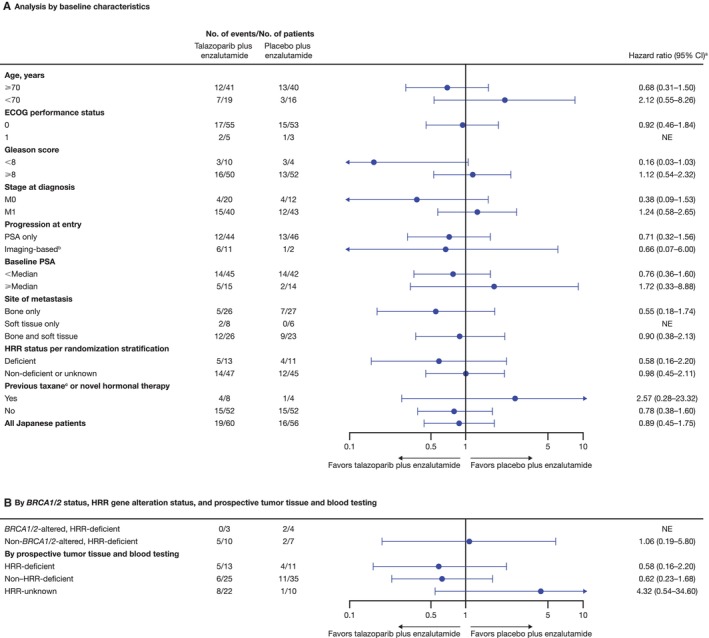
rPFS by BICR by (A) baseline characteristics and (B) *BRCA1/2* gene alteration status, and HRR gene alteration status (Japanese subgroup of the all‐comers intent‐to‐treat population). BICR, blinded independent central review; CI, confidence interval; ECOG, Eastern Cooperative Oncology Group; HRR, homologous recombination repair; M0, nonmetastatic; M1, metastatic; NE, not evaluable; PSA, prostate‐specific antigen; rPFS, radiographic progression‐free survival. ^a^Hazard ratio for all patients was based on a Cox model stratified by the randomization stratification factors. For all subgroups, hazard ratio was based on an unstratified Cox model with treatment as the only covariate. ^b^With or without PSA progression. ^c^All received docetaxel.

In the Japanese subgroup of the all‐comers population, median rPFS was not reached (NR) in either arm (talazoparib arm, NR; 95% CI, 27.9 months–NR; placebo arm, NR; 95% CI, 24.9 months–NR) with an HR of 0.89 (95% CI, 0.45–1.75; Figure 1A). As previously reported [6], median rPFS for the overall all‐comers population was NR (95% CI, 27.5 months–NR) in the talazoparib arm and 21.9 months (95% CI, 16.6–25.1) in the placebo arm (HR = 0.63; 95% CI, 0.51–0.78; *p* < 0.0001; Figure 1B). A greater proportion of patients in the Japanese subgroup compared with the overall all‐comers population were receiving ongoing treatment without an rPFS event at data cutoff (Figure 1). Investigator‐assessed rPFS in the Japanese subgroup had an HR of 0.62 (95% CI, 0.28–1.35; median NR in either arm) (Figure S2A; investigator‐assessed rPFS in the overall all‐comers population is shown in Figure S2B). rPFS by BICR and selected baseline characteristics or gene alteration status in the Japanese subgroup is shown in Figure 2A,B, respectively; results should be interpreted with caution given the small sample size in some subgroups.

In the HRR‐deficient population, treatment with talazoparib plus enzalutamide resulted in a 38% reduced risk of rPFS in the Japanese subgroup (HR = 0.62; 95% CI, 0.22–1.71) and a 55% reduced risk in the overall HRR‐deficient population (HR = 0.45; 95% CI, 0.33–0.61; *p* < 0.0001) versus placebo plus enzalutamide (Figure S3A,B). Among patients with *BRCA1/2* gene alterations in the HRR‐deficient population, rPFS favored talazoparib plus enzalutamide over placebo plus enzalutamide in the overall HRR‐deficient population (HR = 0.20; 95% CI, 0.11–0.36; *p* < 0.0001), with a similar trend in the Japanese subgroup, despite small patient numbers (*n* = 10; HR < 0.01; 95% CI, < 0.01–NR; Figure S4A,B). Among patients without *BRCA1/2* gene alterations in the HRR‐deficient population, rPFS favored talazoparib plus enzalutamide versus placebo plus enzalutamide in the overall HRR‐deficient population (HR = 0.68; 95% CI, 0.46–1.02; *p* = 0.060), with a similar trend in the Japanese subgroup (*n* = 29; HR = 0.92; 95% CI, 0.28–3.07; Figure S5A,B).

In the all‐comers population and at this data cutoff (August 16, 2022), OS data remain immature for both the Japanese subgroup (30% and 23% of patients experienced an OS event in the talazoparib and placebo arms, respectively) and the overall all‐comers population (31% and 32%, respectively; Figure S6A,B). Median duration of follow‐up for OS was 30.4 and 28.6 months in the Japanese subgroup and 28.0 and 27.1 months in the overall all‐comers population in the talazoparib and the placebo arms, respectively. At the interim analysis, the HR for death was 1.06 in the Japanese subgroup (95% CI, 0.51–2.20) and 0.89 (95% CI, 0.69–1.14; *p* = 0.35) in the overall all‐comers population. The HR for PFS2 was 0.86 (95% CI, 0.44–1.70) in the Japanese subgroup and 0.77 (95% CI, 0.61–0.98) in the overall all‐comers population in favor of talazoparib plus enzalutamide.

**FIGURE 3:**
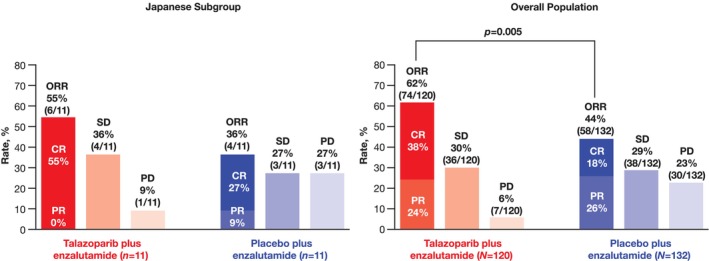
Objective response rate by BICR (all‐comers population with measurable disease at baseline). BICR, blinded independent central review; CR, complete response; ORR, objective response rate; PD, progressive disease; PR, partial response; SD, stable disease.

ORR in patients with measurable disease at baseline was similar between the Japanese subgroup and the overall all‐comers population (Figure 3). ORR in the talazoparib arm was 55% (*n* = 6/11; all were complete responses [CR]) in the Japanese subgroup and 62% (*n* = 74/120) in the overall all‐comers population (CR in 38%). In the placebo arm, ORR was 36% (*n* = 4/11; CR in 27%) in the Japanese subgroup and 44% in the overall all‐comers population (*n* = 58/132; CR in 18%).

**FIGURE 4:**
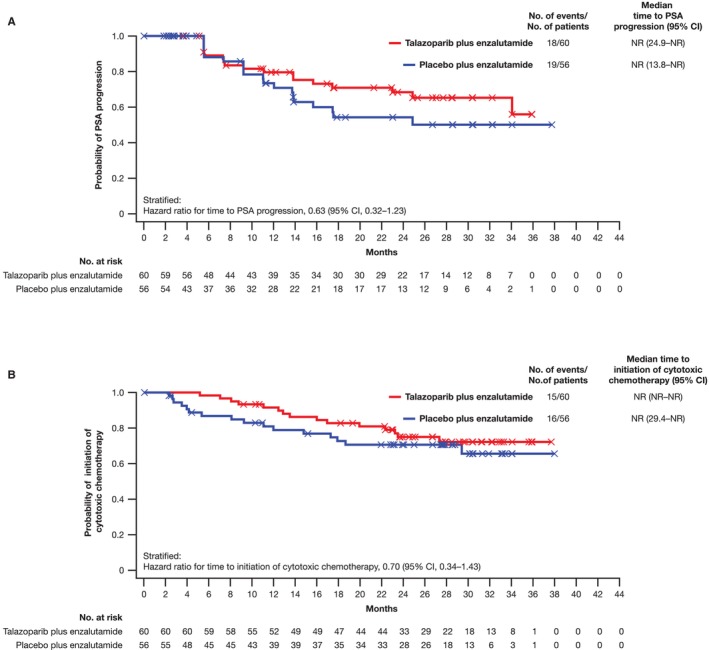
(A) Time to PSA progression and (B) time to initiation of cytotoxic chemotherapy (Japanese subgroup of the all‐comers intent‐to‐treat population). CI, confidence interval; NR, not reached; PSA, prostate‐specific antigen.

The HR for time to PSA progression in the Japanese subgroup was 0.63 (95% CI, 0.32–1.23), with the median time NR (95% CI, 24.9 months–NR) for the talazoparib arm and NR (95% CI, 13.8 months–NR) in the placebo arm (Figure 4A). For time to initiation of cytotoxic chemotherapy, the HR in the Japanese subgroup was 0.70 (95% CI, 0.34–1.43), with the median time NR (95% CI, NR–NR) for the talazoparib arm and NR (95% CI, 29.4 months–NR) in the placebo arm (Figure 4B).

### 3.3 Safety

A summary of treatment‐emergent adverse events (TEAEs) in the Japanese subgroup and the overall all‐comers population is shown in Table 2. Below we present results for the talazoparib arm. In the Japanese subgroup, 33% experienced serious adverse events compared with 39% in the overall all‐comers population. Grade 5 TEAEs occurred in 2 (3%) patients in the Japanese subgroup and 13 (3%) patients in the overall all‐comers population; no grade 5 TEAEs in the talazoparib arm were considered treatment related.

**TABLE 2:** Summary of TEAEs and dose modification and discontinuation due to AE (all‐comers safety population).

	Japanese subgroup		Overall all‐comers population	
TEAEs, *n* (%)	Talazoparib + enzalutamide (*n* = 60)	Placebo + enzalutamide (*n* = 56)	Talazoparib + enzalutamide (*N* = 398)	Placebo + enzalutamide (*N* = 401)
**Any TEAE**	60 (100)	49 (88)	392 (98)	379 (95)
Treatment‐related	58 (97)	32 (57)	357 (90)	279 (70)
**SAEs**	20 (33)	9 (16)	157 (39)	107 (27)
Treatment‐related	7 (12)	1 (2)	78 (20)	12 (3)
**Grade 3/4 TEAEs**	50 (83)	17 (30)	286 (72)	163 (41)
**Grade 5 TEAEs**	2 (3)	0	13 (3)	18 (4)
Treatment‐related	0	0	0	2 (< 1)^a^
**Dose interruption of talazoparib or placebo due to AE**	48 (80)	13 (23)	247 (62)	84 (21)
**Dose reduction of talazoparib or placebo due to AE**	41 (68)	3 (5)	210 (53)	27 (7)
**Discontinuation of talazoparib or placebo due to AE**	16 (27)	4 (7)	75 (19)	49 (12)

Abbreviations: AE, adverse event; SAE, serious adverse event; TEAE, treatment‐emergent adverse event.

^a^ One patient experienced grade 5 SARS‐CoV‐2 and one patient experienced death of unknown cause. In both cases the investigators considered that there was a reasonable possibility the event was related to study treatments.

**FIGURE 5:**
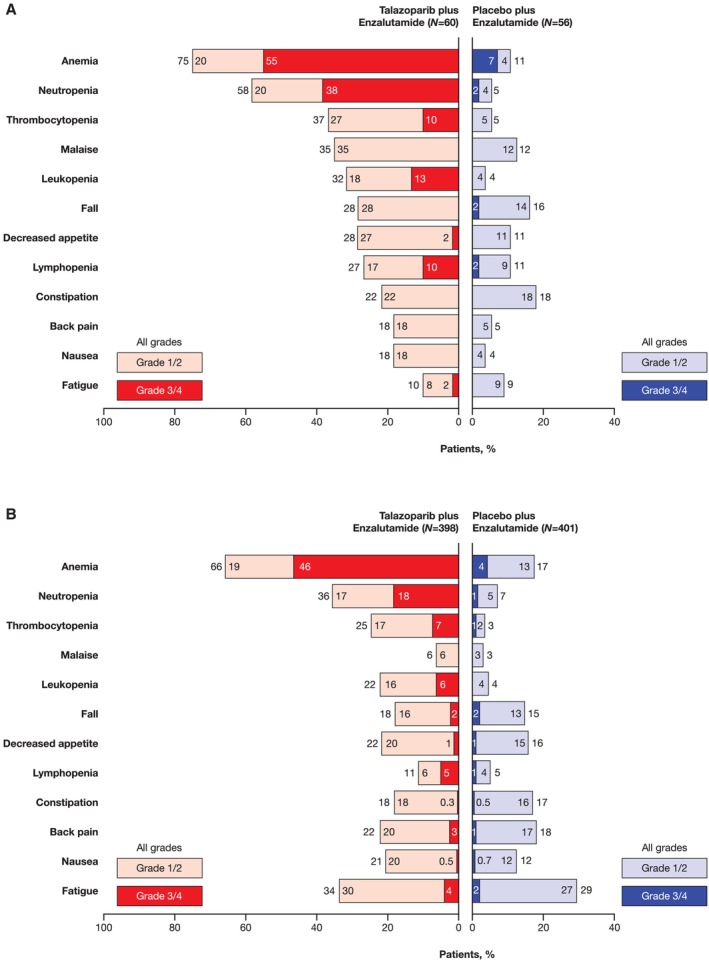
Summary of the most common (≥ 20% in either treatment arm in the Japanese or overall all‐comers safety population) all‐causality TEAEs in the (A) Japanese subgroup and (B) overall all‐comers population. TEAE, treatment‐emergent adverse event. Incidence of TEAEs by all grades, grade 1/2, and grade 3/4 have been independently rounded to whole numbers or to one decimal place if < 1.

**FIGURE 6:**
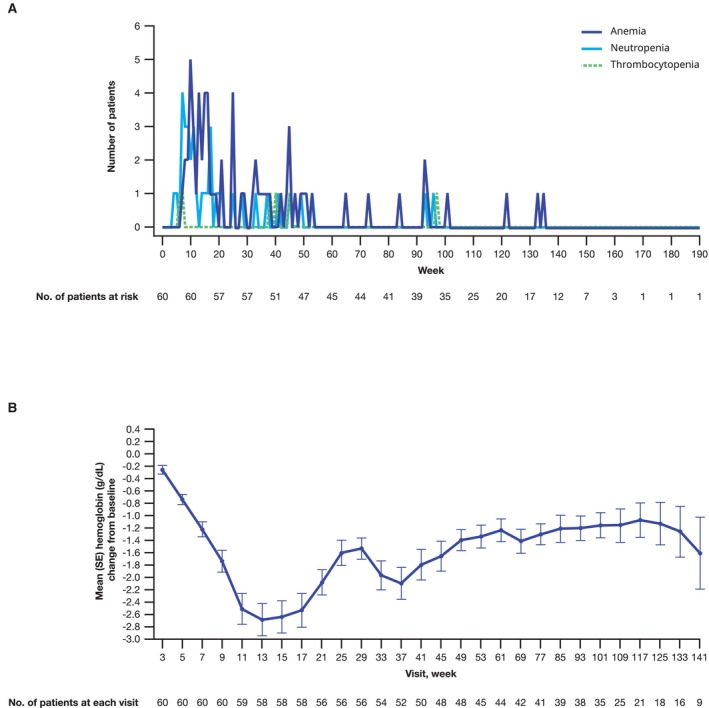
(A) Incidence of grade 3/4 hematologic TEAEs^a^ and (B) change in hemoglobin from baseline in the talazoparib plus enzalutamide arm (Japanese subgroup of the all‐comers safety population). MedDRA, Medical Dictionary for Regulatory Activities; SE, standard error; TEAE, treatment‐emergent adverse event. ^a^Within each week, patients with new reports of TEAEs within the clustered preferred term were counted. MedDRA v25.0 coding dictionary applied. Preferred terms clustered for anemia (anemia, hematocrit decreased, hemoglobin decreased, red blood cell count decreased), neutropenia (neutropenia, neutrophil count decreased), and thrombocytopenia (thrombocytopenia, platelet count decreased).

The most common any‐grade all‐causality TEAEs in the all‐comers Japanese subgroup were myelosuppression related: anemia (75%), neutropenia (58%), thrombocytopenia (37%), and malaise (35%; Figure 5). In the overall all‐comers population, the most common any‐grade all‐causality TEAEs were anemia (66%), neutropenia (36%), fatigue (34%), and thrombocytopenia (25%; Figure 5). The most common grade 3/4 TEAEs in the Japanese subgroup were anemia (55%), neutropenia (38%), leukopenia (13%), thrombocytopenia (10%), and lymphopenia (10%). The AE profile was similar in the overall all‐comers population (Figure 5). The incidence of grade 3/4 hematologic events by week is shown in Figure 6A. In the Japanese subgroup, median time to onset of first grade ≥ 3 hematologic events was 3.3 months for anemia, 2.1 months for neutropenia, and 9.0 months for thrombocytopenia. Anemia was not a cumulative drug‐related toxicity. The greatest reduction from baseline hemoglobin levels occurred at 13 weeks of treatment (Figure 6B).

The rates of talazoparib dose interruption, reduction, and discontinuation in the Japanese subgroup were 80%, 68%, and 27%, respectively, and 62%, 53%, and 19%, respectively, in the overall all‐comers population (Table 2). The most common TEAEs leading to a discontinuation of talazoparib in the Japanese subgroup and the overall all‐comers population, respectively, were anemia (12% and 8%), neutropenia (5% and 3%), and thrombocytopenia (0% and 0.5%). Similarly, the most common TEAEs leading to a dose reduction of talazoparib in the Japanese subgroup and the overall all‐comers population, respectively, were anemia (50% and 43%), neutropenia (32% and 15%), and thrombocytopenia (7% and 6%). Despite dose modifications, the median relative dose intensity and duration of treatment of talazoparib was 72% and 98 weeks, respectively, in the Japanese subgroup (overall all‐comers population: 84% and 86 weeks).

Hematologic TEAEs were managed with packed red blood cell transfusions and dose modifications. In the Japanese subgroup and the overall all‐comers population, respectively, 38% and 39% of patients received packed red blood cell transfusions. At least one hematologic supportive treatment was received by 10% of patients in the Japanese subgroup and 13% in the overall all‐comers population. Erythropoietin‐stimulating agents, granulocyte‐stimulating factors, and platelet‐stimulating factors were received by 0%, 10%, and 0% of patients in the Japanese subgroup, respectively, and 8%, 8%, and 2% in the overall all‐comers population, respectively.

### 3.4 Pharmacokinetics

**FIGURE 7:**
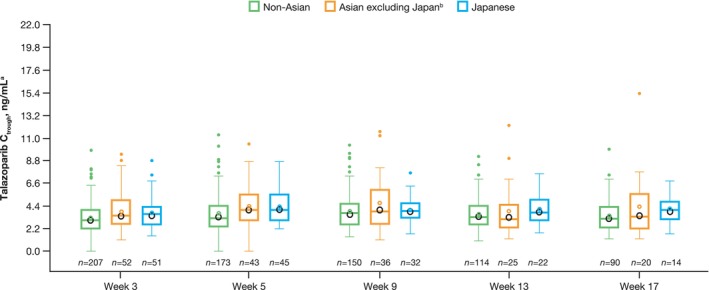
Talazoparib *C*_trough_ in Japanese, Asian, and non‐Asian patients (all‐comers pharmacokinetics evaluable population). *C*_trough_, plasma concentration at predose in patients following talazoparib 0.5 mg QD and enzalutamide 160 mg; QD, once daily. Closed circles represent individual participant values, open‐colored circles represent the mean, and black circles represent the geometric mean. For geometric mean, zero values have been removed prior to log transformation. Box plots provide the median, first, and third quartiles with whiskers to the last point within 1.5 times the interquartile range. The remaining summary statistics have been calculated using zero values. ^a^The lower limit of quantification is 0.025 ng/mL; concentrations below this value have been set to zero. ^b^Includes patients enrolled in Republic of Korea and China.

The predose plasma drug concentration of talazoparib (*C*_trough_) among patients receiving talazoparib plus enzalutamide was generally comparable between Japanese, Asian (China and Republic of Korea, excluding Japan), and non‐Asian patients (Table S1 and Figure 7).

## 4 Discussion

In the large, randomized TALAPRO‐2 study involving patients with mCRPC, talazoparib plus enzalutamide resulted in a clinically meaningful improvement in rPFS by BICR versus placebo plus enzalutamide in the overall all‐comers population (HR = 0.63; 95% CI, 0.51–0.78; median NR vs. 21.9 months in the placebo arm) [6]. In the Japanese subgroup, there was a trend favoring the combination arm; however, the median rPFS by BICR was NR in either treatment arm (HR = 0.89; 95% CI, 0.45–1.75). As shown by baseline disease characteristics, Japanese patients had a better prognosis, which may explain why rPFS was NR in the placebo arm of this subgroup. Differences in baseline characteristics may have influenced the efficacy results. Fewer Japanese patients had received prior docetaxel in the castration‐sensitive setting than in the overall all‐comers population, likely because docetaxel was approved for treatment of metastatic castration‐sensitive prostate cancer in Japan in 2021 [22], partway through TALAPRO‐2, and the median baseline PSA among Japanese patients was lower than in the overall all‐comers population. There were also more censored observations in the Japanese subgroup than in the overall all‐comers population, which would impact the rPFS results. Furthermore, a higher proportion of patients in the talazoparib arm of the Japanese subgroup were still ongoing treatment without an event at the time of analysis compared with the placebo arm regardless of HRR gene alteration status. Because of its exploratory nature, this analysis of the Japanese subgroup from TALAPRO‐2 is limited by lack of formal statistical power, which restricts our ability to draw stronger conclusions on the statistical significance of efficacy benefits with talazoparib plus enzalutamide compared with placebo plus enzalutamide.

rPFS by investigator assessment in the Japanese subgroup also favored the combination arm (HR = 0.62; 95% CI, 0.28–1.35), with the median time NR for either treatment arm. rPFS results observed for Japanese patients in the HRR‐deficient population suggest benefit with combination treatment for patients with HRR gene alterations, including the *BRCA1/2*‐deficient subgroup. In the all‐comers population, Japanese patients who received talazoparib plus enzalutamide also showed improvements in all secondary endpoints: ORR, PFS2, time to PSA progression, and time to initiation of cytotoxic chemotherapy versus placebo plus enzalutamide. The combination of talazoparib with enzalutamide resulted in an ORR of 55%, with all patients having a CR, implying benefit from PARPi and ARSi combination therapy. OS data are immature and with too few events in the Japanese subgroup to interpret the results.

The phase 3 PROfound trial (NCT02987543) evaluated olaparib monotherapy compared with abiraterone or enzalutamide in patients with mCRPC and HRR gene alterations whose disease had progressed on prior next‐generation hormonal therapy [23]. An analysis of the HRR‐deficient Asian subgroup from PROfound (*n* = 101; Japanese patients, *n* = 57) demonstrated a trend for improved rPFS for patients who received olaparib and a comparable safety profile with the overall global population [24]. These data are consistent with the results of this analysis where we also found similar efficacy between the Japanese subgroup and the overall HRR‐deficient population who received talazoparib plus enzalutamide.

Frequencies of HRR gene alterations, including *BRCA1/2* gene alterations, were similar between the Japanese subgroup and the overall all‐comers population. Six percent of Japanese patients had a *BRCA2* gene alteration, which is similar to the rate observed among Japanese patients in the PROfound trial (8%) [24, 25]. This finding suggests that the sample of Japanese patients with mCRPC in TALAPRO‐2 reflects the background rate of HRR gene alterations in this population [25].

Compared with the previously published safety data in the overall all‐comers population [6], no new safety signals were identified for the Japanese subgroup. At baseline, grade 1/2 anemia was present in 63% and 49% of patients in the talazoparib arm of the Japanese subgroup and the overall all‐comers population, respectively. On‐target anemia was the most common grade 3/4 TEAE in the talazoparib arm in both the Japanese subgroup and the overall all‐comers populations. These results are consistent with a previous study involving a small number of Japanese patients (*N* = 9) with solid tumors who received talazoparib monotherapy and found that hematologic AEs (anemia, neutropenia, and thrombocytopenia) were among the most common TEAEs (each 22%) [16]. Grade 3/4 neutropenia had the greatest difference in incidence between the talazoparib arm of the Japanese subgroup and the overall all‐comers population (38% and 18%, respectively). This result may be expected given prior reports that Asian patients may develop neutropenia at higher rates compared with non‐Asian patients in response to certain systemic cancer therapies [14]. Despite the higher incidence, only 5% of Japanese patients discontinued talazoparib due to neutropenia. Hematologic TEAEs were generally managed in the Japanese subgroup through close monitoring of patients, dose modifications, and hematologic supportive care measures, including transfusion. The time to onset of grade 3/4 hematologic AEs in the talazoparib arm and the course of hemoglobin change in Japanese patients were consistent with those in the overall all‐comers population [26].

Talazoparib *C*_trough_ was similar between Japanese patients, non‐Japanese Asian patients, and non‐Asian patients. These data demonstrate that, despite differences in median dose intensity, 0.5 mg talazoparib in combination with enzalutamide provides a similar level of steady‐state talazoparib exposure for Japanese patients compared with the overall all‐comers population. These results provide reassurance that the safety profile presented here for the Japanese subgroup is based on a similar level of talazoparib exposure as the overall population. In addition, the results are consistent with two prior phase 1 pharmacokinetic studies of talazoparib monotherapy, which demonstrated that talazoparib maximum observed plasma concentration (*C*_max_) and area under the plasma concentration–time curve (AUC) were similar between Japanese and non‐Japanese patients [15, 16].

The aforementioned pharmacokinetic data showed similar talazoparib pharmacokinetics in Japanese and non‐Japanese patients. The increased incidence of grade 3/4 neutropenia in Japanese patients relative to the overall all‐comers population may therefore be explained by differences in other factors, including weight and baseline absolute neutrophil counts. Lower weight and lower baseline absolute neutrophil counts have been shown to be prognostic of grade ≥ 3 neutropenia in previous exposure‐safety analyses of the TALAPRO‐2 study, and the EMBRACA and ABRAZO studies [27, 28]. Both body weight and baseline neutrophil counts are generally lower in Asian compared with non‐Asian populations [29, 30].

The 2023 Japanese Clinical Practice Guidelines for Prostate Cancer currently recommend first‐line treatment with androgen deprivation therapy plus an ARSi or docetaxel for patients with mCRPC [31]. While these guidelines state that olaparib monotherapy may be considered in patients with *BRCA1/2* gene alterations, the recommendation level is “weak” [31]. However, with recent approvals in Japan of both talazoparib plus enzalutamide (in 2024) [10] and olaparib plus abiraterone (in 2023) [32] in patients with *BRCA1/2* gene alterations, PARPi plus ARSi combinations are emerging as a treatment option for patients with mCRPC. Data from the current TALAPRO‐2 analysis showing efficacy benefit and a consistent safety profile with talazoparib plus enzalutamide in Japanese patients with *BRCA1/2* gene alterations support the consideration of this combination as an additional first‐line therapy option for these patients.

## 5 Conclusions

This exploratory, subgroup analysis of Japanese patients in TALAPRO‐2 who received talazoparib plus enzalutamide demonstrated efficacy outcomes consistent with those seen in the overall all‐comers population. In addition, the safety profile and pharmacokinetics of the combination therapy were similar between Japanese patients and the overall all‐comers population. Final overall survival data and additional long‐term safety follow‐up will provide further information on the clinical benefit in patients with and without tumor HRR gene alterations in both the Japanese subgroup and the overall population.

## Author Contributions

**Nobuaki Matsubara:** Investigation, Writing – review & editing. **Hideaki Miyake:** Investigation, Writing – review & editing. **Hiroji Uemura:** Investigation, Writing – review & editing. **Atsushi Mizokami:** Investigation, Writing – review & editing. **Hiroaki Kikukawa:** Investigation, Writing – review & editing. **Takeo Kosaka:** Investigation, Writing – review & editing. **Kazuo Nishimura:** Investigation, Writing – review & editing. **Motonobu Nakamura:** Investigation, Writing – review & editing. **Kazuki Kobayashi:** Investigation, Writing – review & editing. **Atsushi Komaru:** Investigation, Writing – review & editing. **Yuko Mori:** Conceptualization, Writing – review & editing. **Shigeyuki Toyoizumi:** Conceptualization, Formal analysis, Writing – review & editing. **Natsuki Hori:** Validation, Conceptualization, Writing – review & editing. **Yoshiko Umeyama:** Writing – review & editing. **Hirotsugu Uemura:** Investigation, Writing – review & editing.

## Ethics Statement

TALAPRO‐2 is being conducted in accordance with the International Ethical Guidelines for Biomedical Research Involving Human Subjects, Good Clinical Practice guidelines, the principles of the Declaration of Helsinki, and local laws. The protocol and amendments were approved by the institutional review board and independent ethics committee for each site.

## Consent

All patients provided written informed consent.

## Conflicts of Interest

Nobuaki Matsubara reports honoraria (personal) from Sanofi; research funding (institution) from Amgen, Astellas Pharma, AstraZeneca, Bayer, Chugai Pharma, Eisai, Janssen, Lilly, MSD, Pfizer, PRA Health Science, Roche, Seagen, Taiho, and Takeda; and travel, accommodations, and/or expenses (personal) from Pfizer. Hideaki Miyake reports honoraria from Astellas Pharma, AstraZeneca, Bayer, Eisai, Ferring, Janssen, Merck, MSD, Nippon Shinyaku, Ono, Pfizer, Sanofi, and Takeda. Hiroji Uemura reports honoraria from Astellas Pharma, AstraZeneca, Bayer, Ferring, Janssen, Merck, MSD, Ono, Pfizer, Sanofi, Chugai, Novartis, Daiichi Sankyo, Nihon Medi‐Physics, Nippon Shinyaku, and Takeda. Atsushi Mizokami reports honoraria from Janssen, Sanofi, Bayer, Takeda, and PDR Pharma. Hiroaki Kikukawa, Takeo Kosaka, Motonobu Nakamura, Kazuki Kobayashi, and Atsushi Komaru report no conflicts of interest. Kazuo Nishimura reports honoraria from Astellas Pharma, AstraZeneca, Bayer, Janssen, MSD, and Merck. Yuko Mori, Shigeyuki Toyoizumi, Natsuki Hori, and Yoshiko Umeyama are employees of Pfizer R&D Japan and may hold stock in Pfizer, Inc. Hirotsugu Uemura reports receiving research grants from Astellas Pharma, AstraZeneca, Chugai, Janssen, Ono, Osaka Urology Research Foundation, and Takeda; consulting fees from BMS, Ono, and Sanofi; lecture and speaker's fees from Bayer, BMS, Janssen, MSD, Takeda, Sanofi, and Pfizer.

## Permission To Reproduce Content

Reprinted from Fizazi et al. December 4, 2023. First‐line talazoparib with enzalutamide in HRR‐deficient metastatic castration‐resistant prostate cancer: the phase 3 TALAPRO‐2 trial. *Nature Medicine*. 2024;30:257–264.

## Supporting information


Data S1.

## Data Availability

Upon request, and subject to review, Pfizer will provide the data that support the findings of this study. Subject to certain criteria, conditions, and exceptions, Pfizer may also provide access to the related individual deidentified participant data. See https://www.pfizer.com/science/clinical‐trials/trial‐data‐and‐results for more information.
