# Supplementary material for: Phase 3 Study of Talazoparib Plus Enzalutamide Versus Placebo Plus Enzalutamide as First‐Line Treatment in Patients With Metastatic Castration‐Resistant Prostate Cancer: TALAPRO‐2 Japanese Subgroup Analysis
Source: Cancer Med. 2024 Dec 31;14(1):e70333. doi: 10.1002/cam4.70333 (PMC11686335; doi:10.1002/cam4.70333)
Supplement: Supplementary file 1 — Data S1. [file CAM4-14-e70333-s001.pdf]

SUPPLEMENTARY TABLE 1 Summary of plasma talazoparib C_{trough} by race and visit[a] (all-comers pharmacokinetics evaluable population).

Race	Parameter	Week 3	Week 5	Week 9	Week 13	Week 17
**Japanese**	*n*	51	45	32	22	14
	Geometric mean (ng/mL) (geometric CV, %)	3.44 (43)	4.03 (38)	3.83 (32)	3.82 (39)	3.84 (41)
**Asian excluding Japan**[b]	*n*	52	43	36	25	20
	Geometric mean (ng/mL) (geometric CV, %)	3.42 (53)	4.01 (51)	4.00 (63)	3.27 (64)	3.46 (77)
**Non-Asian**	*n*	207	173	150	114	90
	Geometric mean (ng/mL) (geometric CV, %)	2.99 (45)	3.34 (52)	3.58 (42)	3.35 (41)	3.17 (42)

C_{trough}, plasma concentration at pre-dose in patients following talazoparib 0.5 mg QD and enzalutamide 160 mg QD; CV, coefficient of variation; *n*, number of patients; QD, once daily.

[a]The lower limit of quantification is 0.025 ng/mL; concentrations below this value have been set to zero.

[b]Includes patients enrolled in Republic of Korea and China.

SUPPLEMENTARY FIGURE 1 TALAPRO-2 study design.

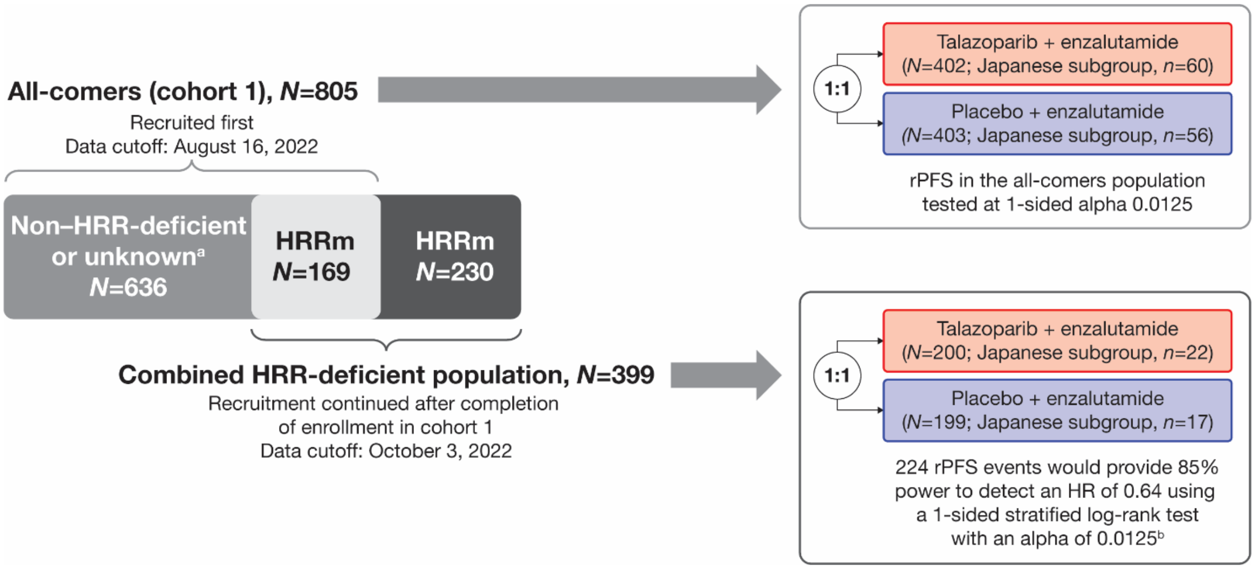

This figure is modified from Fizazi et al. 04 December 2023. First-line talazoparib with enzalutamide in HRR-deficient metastatic castration-resistant prostate cancer: the phase 3 TALAPRO-2 trial. *Nature Medicine* 2024;30:257-264. This figure is licensed under a Creative Commons Attribution 4.0 International License. To view a copy of this license, visit http://creativecommons.org/licences/by/4.0/.

HR, hazard ratio; HRR, homologous recombination repair; HRRm, positive for alterations in homologous recombination repair genes; rPFS, radiographic progression-free survival.

[a]In the all-comers population, 27% of patients in the talazoparib plus enzalutamide and 25% in the placebo plus enzalutamide arms were of unknown HRR gene alteration status. In the combined HRR-deficient population, 3 patients (1, talazoparib plus enzalutamide; 2, placebo plus enzalutamide) did not have HRR gene alterations and 1 patient in the talazoparib arm was of unknown HRR gene alteration status.

[b]An interim analysis (IA) was planned with approximately 70% of the total required events. The HRRm cohort would be stopped for efficacy if the prespecified efficacy boundary was crossed ($p \leq 0.003$). As the efficacy boundary was crossed at the IA rPFS, this became the final analysis. Survival and safety follow-up is continuing. All other endpoints are final.

SUPPLEMENTARY FIGURE 2 rPFS by investigator: (A) Japanese subgroup; (B) overall population (all-comers intent-to-treat population).

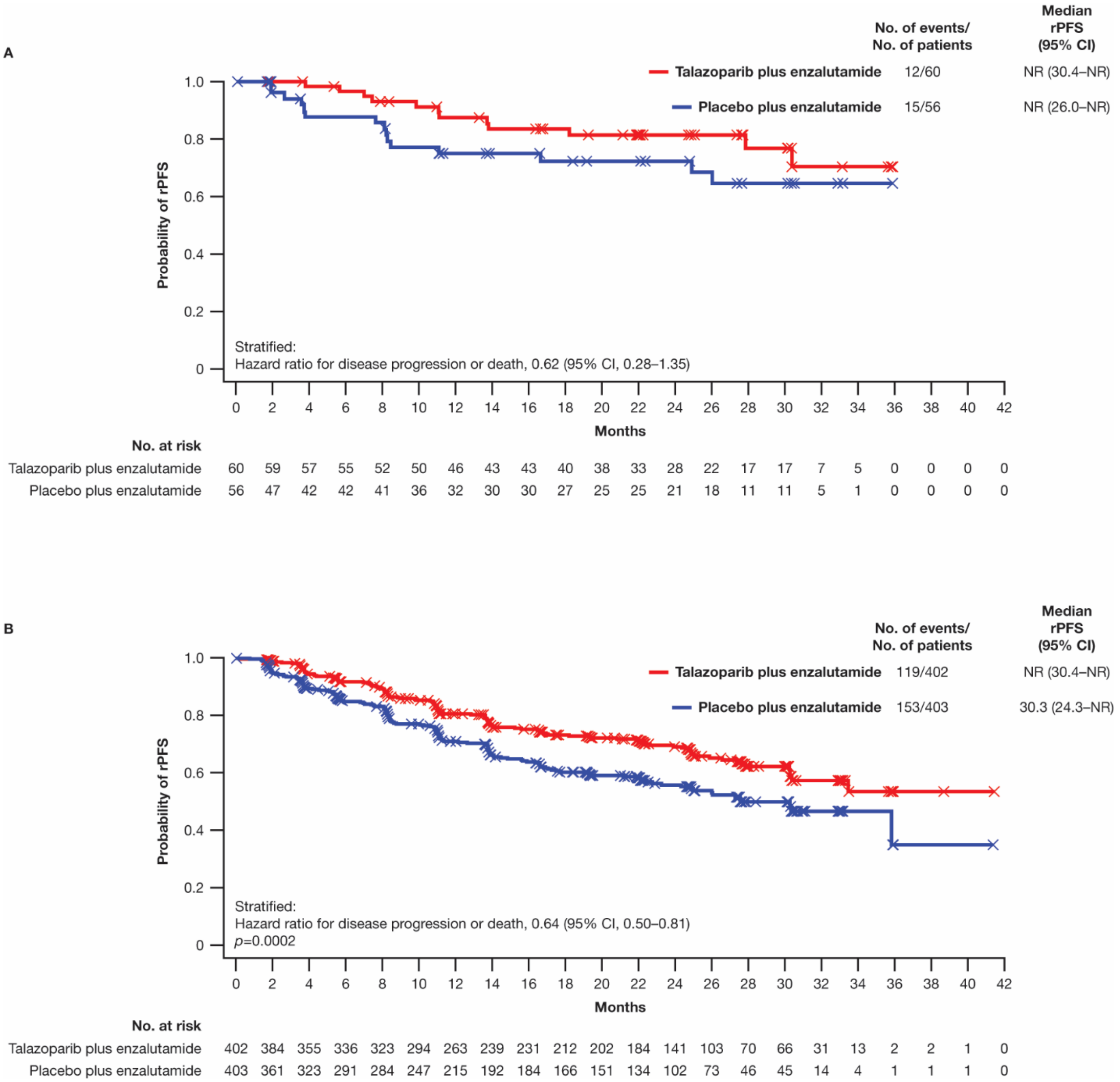

CI, confidence interval; NR, not reached; rPFS, radiographic progression-free survival. Figure panel B is reprinted from *The Lancet*, Volume 402, Agarwal et al., Talazoparib plus enzalutamide in men with first-line metastatic castration-resistant prostate cancer (TALAPRO-2): a randomised, placebo-controlled, phase 3 trial, pages 291-303, 2023, with permission from Elsevier.

SUPPLEMENTARY FIGURE 3 rPFS by BICR: (A) Japanese subgroup; (B) overall population (HRR-deficient intent-to-treat population).

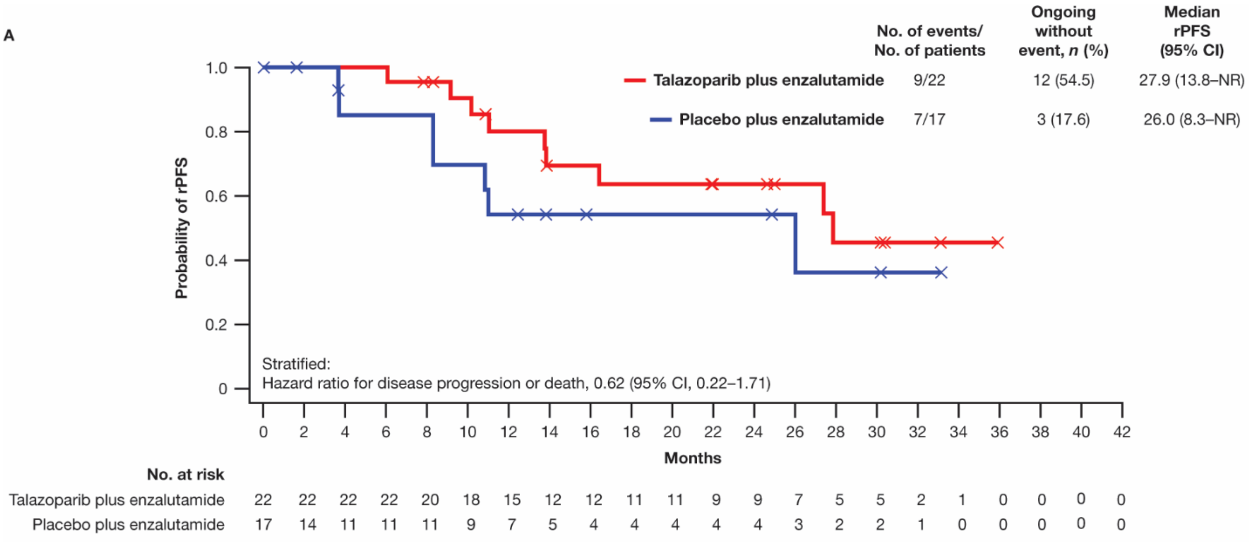

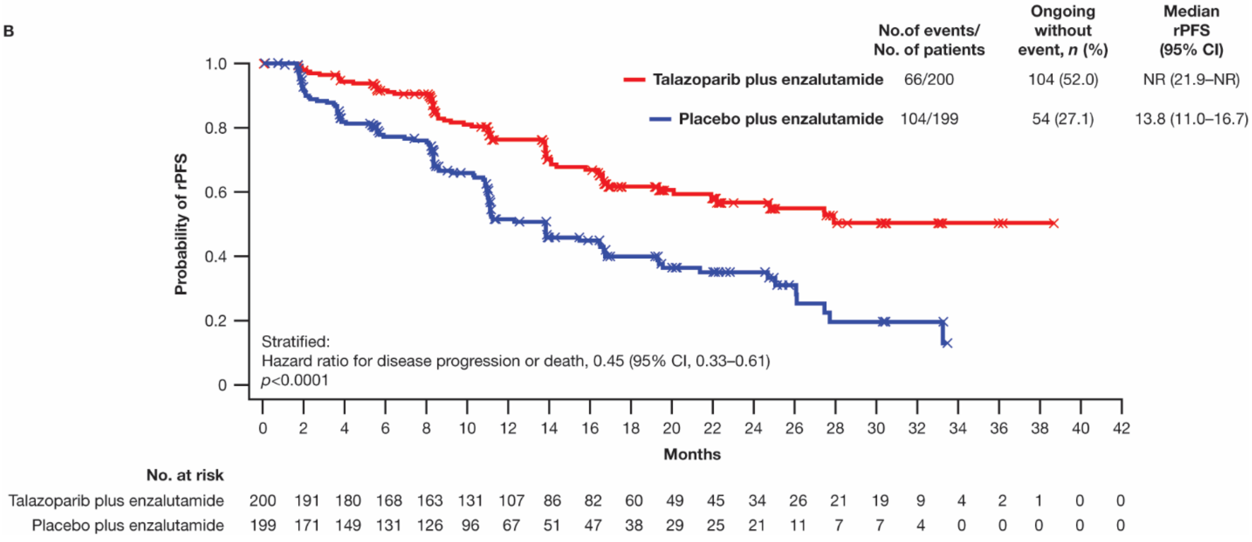

BICR, blinded independent central review; CI, confidence interval; NR, not reached; HRR, homologous recombination repair; rPFS, radiographic progression-free survival.

Figure panel B is modified from Fizazi et al. 04 December 2023. First-line talazoparib with enzalutamide in HRR-deficient metastatic castration-resistant prostate cancer: the phase 3 TALAPRO-2 trial. *Nature Medicine*. 2024;30:257-264. This figure is licensed under a Creative Commons Attribution 4.0 International License. To view a copy of this license, visit https://creativecommons.org/licenses/by/4.0/.

SUPPLEMENTARY FIGURE 4 rPFS by BICR in patients with *BRCA1/2* gene alterations: (A) Japanese subgroup; (B) overall population (HRR-deficient intent-to-treat population).

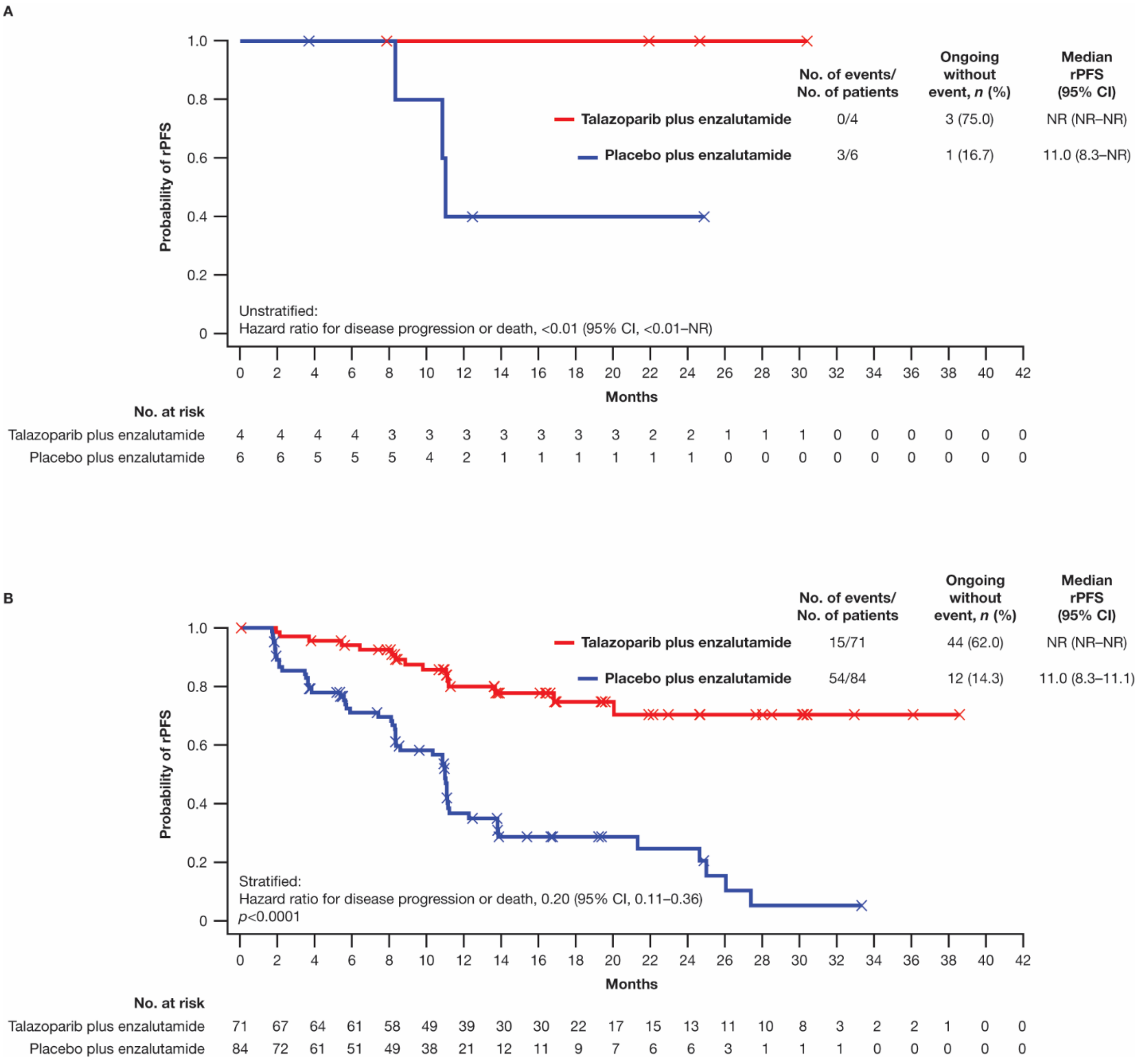

BICR, blinded independent central review; CI, confidence interval; HRR, homologous recombination repair; NR, not reached; rPFS, radiographic progression-free survival.

SUPPLEMENTARY FIGURE 5 rPFS by BICR in patients with non-*BRCA1/2* HRR gene alterations: (A) Japanese subgroup; (B) overall population (HRR-deficient intent-to-treat population).

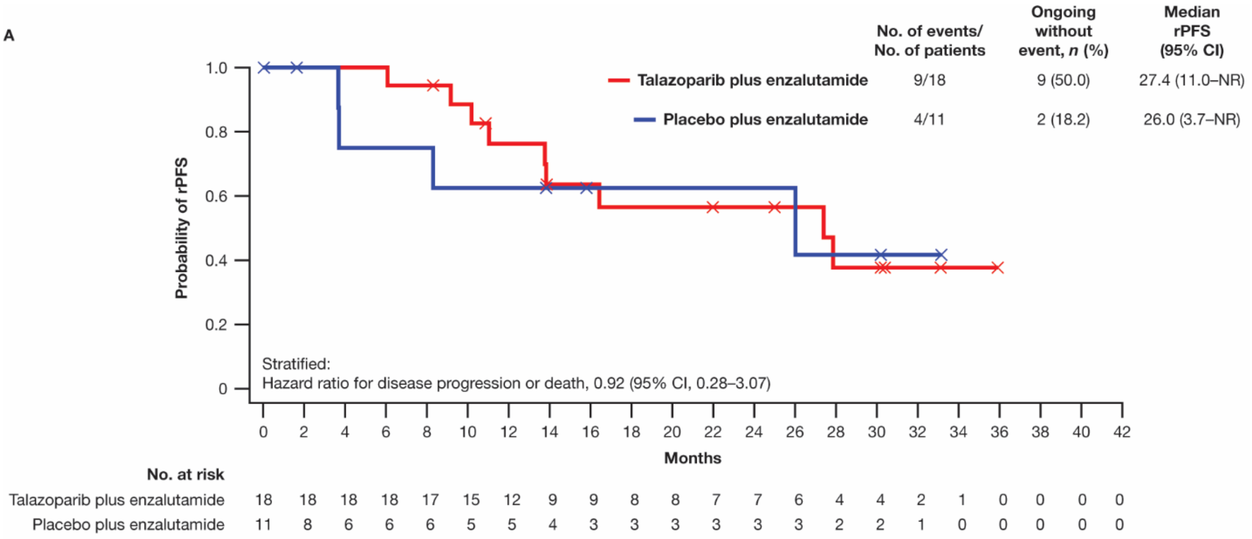

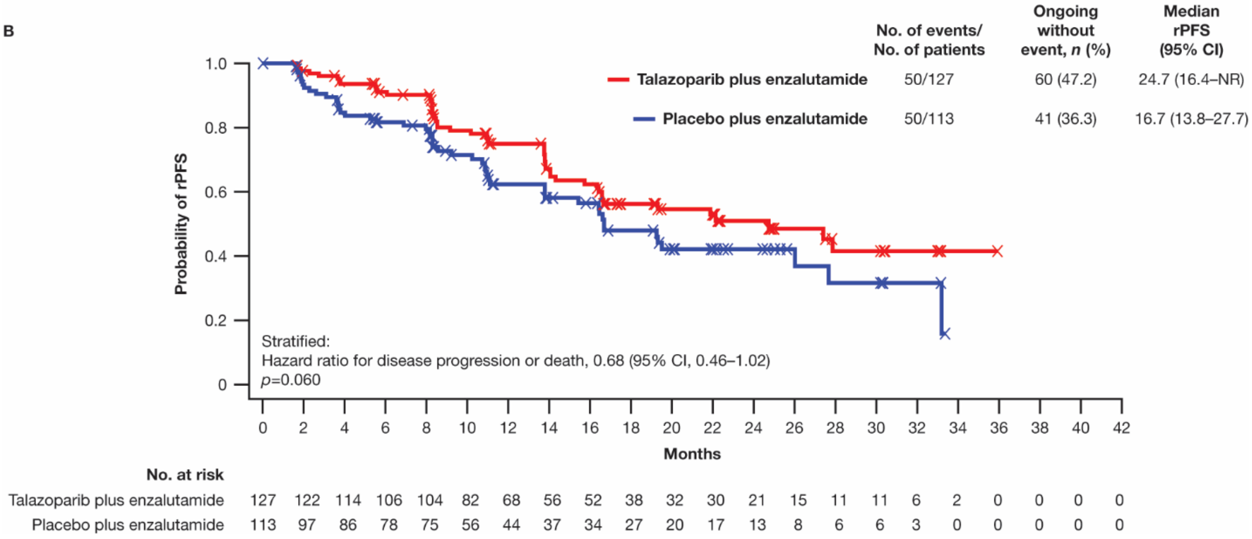

BICR, blinded independent central review; CI, confidence interval; HRR, homologous recombination repair; NR, not reached; rPFS=radiographic progression-free survival.

SUPPLEMENTARY FIGURE 6 Interim analysis of overall survival: (A) Japanese subgroup; (B) overall population (all-comers intent-to-treat population).

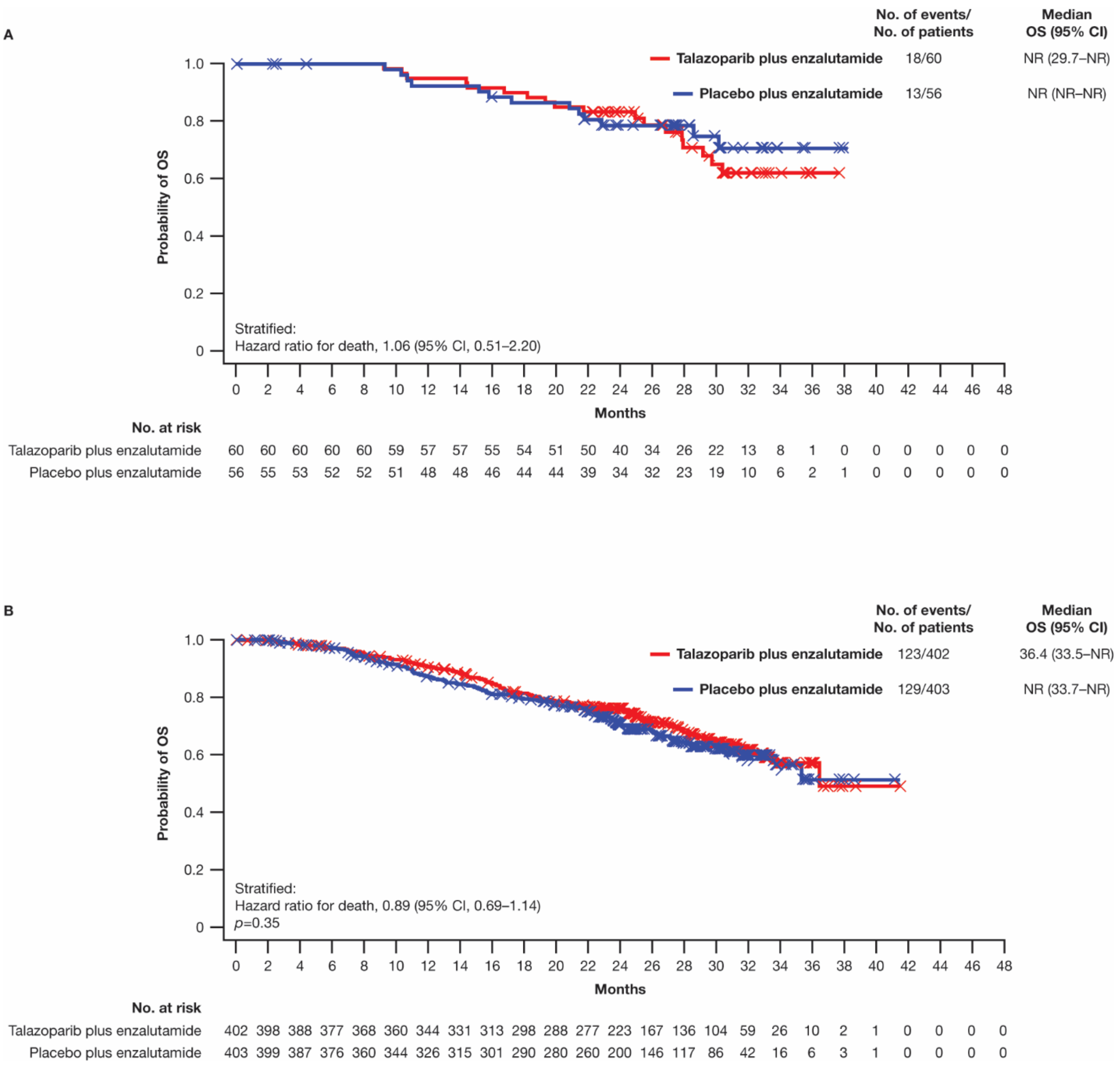

CI, confidence interval; NR, not reached; OS, overall survival.

Panel B is reprinted from *The Lancet*, Volume 402, Agarwal et al., Talazoparib plus enzalutamide in men with first-line metastatic castration-resistant prostate cancer (TALAPRO-2): A randomized, placebo-controlled, Phase 3 trial, pages 291–303, 2023, with permission from Elsevier.
